# Harti Hauora Tamariki: randomised controlled trial protocol for an opportunistic, holistic and family centred approach to improving outcomes for hospitalised children and their families in Aotearoa, New Zealand

**DOI:** 10.3389/fped.2024.1359214

**Published:** 2024-02-22

**Authors:** Nina Scott, Polly E. Atatoa Carr, Amy R. Jones, Peter Sandiford, Bridgette Masters-Awatere, Helen Clark

**Affiliations:** ^1^ Matauranga Māori, Rangahau Hauora Māori, Te Aka Whai Ora, Hamilton, New Zealand; ^2^ Te Whatu Ora Waikato, Hamilton, New Zealand; ^3^ Te Ngira, Institute for Population Research, University of Waikato, Hamilton, New Zealand; ^4^ Te Whatu Ora Waitemata, Auckland, New Zealand; ^5^ School of Psychology, University of Waikato, Hamilton, New Zealand

**Keywords:** child health, indigenous research, health screening, navigators, health inequities, RCT, holistic, Kaupapa Māori

## Abstract

**Background:**

Health and wellbeing inequities between the Indigenous Māori and non-Māori populations in Aotearoa, New Zealand continue to be unresolved. Within this context, and of particular concern, hospitalisations for diseases of poverty are increasing for tamariki Māori (Māori children). To provide hospitalised tamariki Māori, and their whānau (families) comprehensive support, a wellbeing needs assessment; the Harti Hauora Tamariki Tool (The Harti tool) was developed. The purpose of this study is to determine how effective the Harti tool is at identifying wellbeing needs, ensuring the documentation of needs, enabling access to services and improving wellbeing outcomes for tamariki and their whānau.

**Methods:**

The study uses a Kaupapa Māori methodology with qualitative and quantitative methods. Qualitative methods include in-depth interviews with whānau. This paper presents an overview of a randomised, two parallel, controlled, single blinded, superiority trial for quantitative evaluation of the Harti programme, and hospital satisfaction with care survey. Participants will be Māori and non-Māori tamariki/children aged 0–4 years admitted acutely to the paediatric medical wards at Waikato Hospital, Hamilton, Aotearoa New Zealand. They will be randomised electronically into the intervention or usual care group. The intervention group will receive usual care in addition to the Harti programme, which includes a 24-section health needs assessment delivered by trained Māori navigators to whānau during the time they are in hospital. The primary endpoint is the relative risk of an acute hospital readmission in the 30 days following discharge for the intervention group patients compared with control group patients. Secondary outcomes include access and utilisation of preventative health services including: oral health care, general practice enrolment, immunisation, healthy home initiatives, smoking cessation and the Well Child Tamariki Ora universal health checks available free of charge for children in Aotearoa New Zealand.

**Discussion:**

Randomised controlled trials are a gold standard for measuring efficacy of complex multifaceted interventions and the results will provide high quality evidence for implementing the intervention nationwide. We expect that this study will provide valuable evidence for health services and policy makers who are considering how to improve the configuration of paediatric hospital services.

**Trial registration:**

The study is registered with the Australian New Zealand Clinical Trials Registry (ANZCTR), registration number: ACTRN12618001079235.

## 1 Introduction

“*Poipoiā te kākano kia puawai*” or “nurture the seed and it will blossom” (1), which also encapsulates the importance of supporting and caring for infants and children to give them the best opportunities in life.

There is an expectation that hospitals provide the best possible care, especially for children, and the health sector is required to ensure that the wellbeing needs of tamariki (children) and their whānau/families are met (2). However, hospital and health sector staff do not have the tools or systems in place to routinely assess broader determinants of wellbeing (such as housing and income support) in order to provide stronger preventative health and wellbeing support for patients and their whānau (families) (3).

Hospitalisations for diseases of poverty (or those with a social gradient), are increasing for tamariki Māori in Aotearoa New Zealand (NZ) (4, 5). This is driven by a multitude of issues including colonisation, racism, and sequential NZ governments inadequate responses to limited access to the determinants of health for whānau Māori (6). Despite this, there is a paucity of literature on improving hospital care for tamariki Māori, and specifically on opportunities to address the drivers of avoidable hospitalisation and rehospitalisation for tamariki in NZ. The research team attempted to address this lack of action by redeveloping and testing a wellbeing assessment and service provision tool for hospitalized tamariki and their whānau. The team are located in the Te Whatu Ora Waikato health services region which serves approximately 29,000 tamariki Māori aged 0–14 years (4), more than any other region in the country.

As in other parts of Aotearoa, persistent, unfair and unjust inequities in access to material resources and health outcomes exist between communities in the Waikato region of NZ (4, 7). As in other parts of the country, the greatest inequities in resources and outcomes lie between the Indigenous and non-Indigenous populations. This is also true for the paediatric population. More than a quarter of Māori in the Waikato live in the most socioeconomically deprived area (Quintile 5), according to the NZ Deprivation Index (8). In 2013, two in five Māori children lived in households with low equivalised annual household incomes (under $15K) (8). This unequal distribution of wealth is a major driver of inequities in child health outcomes. Even after accounting for unequal distribution of wealth, stark inequities in hospitalisations between tamariki Māori and non-Māori remain. Only ten percent of children aged 0–4 years in Waikato are tamariki Māori, yet tamariki Māori accounted for 44% of hospital admissions for children aged 0–4 years in the winter months of 2014 (9). Of these children, most (71%) were admitted to hospital with a preventable medical condition that had a social gradient, meaning that children from areas of low deprivation had lower disease rates than those from higher deprivation areas (9). These diseases included acute bronchiolitis (43% of the winter admissions in this age group in 2014), pneumonia (11%), asthma (6%) and acute upper respiratory tract infections (4%). Of deep concern is that 51% of tamariki Māori admitted with diseases of poverty were re-hospitalised (at least once) within the next 6 months. At 12 months, for all causes of admission, the risk of readmission for Māori was 56% (9). At the time of the study, in a reported attempt to drive improvements in health spending, health delivery and health equity, a quality improvement target for the NZ Ministry of Health was developed to reduce readmissions for preventable conditions for tamariki aged 0–4 (10).

As a response to these issues of unmet health need and inequitable and unacceptable hospital readmission rates for tamariki Māori in the Waikato, Te Puna Oranga (the Māori Health Unit) at Te Whatu Ora Waikato led a co-design process with nurses, Kaitiaki (cultural support workers) and doctors who had worked or were working in child health at Waikato Hospital (11). The co-design process resulted in the development of a tool to identity health and wellbeing needs, and to support opportunities to address need and enable the provision of holistic and preventative care particularly for hospitalised tamariki Māori and their whānau. The Harti Tool was added in a paper folder to patient notes and used in the paediatric medical wards at Waikato Hospital from 2014 to 2017. The paediatric medical charge nurse reported that the majority of patients and their whānau were provided access to a wellbeing needs assessment using the tool during this time.

The team presented the Harti approach to colleagues around the country at peer meetings, conferences and seminars. The tool was shared freely with all people and groups who requested it. As a result, the tool was implemented in other areas around the country. After the Harti tool had been in place for 3 years, the team decided to apply for a Health Research Council of New Zealand grant to measure the impact of the tool. Another driver was the need to update the screening questions and follow up protocols in the tool and to further develop thinking around the model of care for the tool.

This paper describes the protocol for the quantitative evaluation of the effectiveness of the Harti Tool using a randomized controlled trial (RCT). Objectives of the RCT (the “Harti RCT”) are:

➢ To provide a quantitative measure of the effectiveness of the Harti tool
➢ To determine the level of unmet need identified by the Harti tool, and
➢ To assess the impact of the Harti tool on meeting those needs and achieving improved outcomes for tamariki.

The Harti RCT aims to answer the following research questions compared with usual care;

➢ Does the use of the Harti tool increase documentation of assessment of health and non-health needs?
➢ Does use of the Harti tool result in a lower readmission risk for hospitalised children?
➢ Does the Harti tool improve satisfaction with hospital experience?
➢ Does the Harti tool increase access to and utilisation of wellbeing services?

## 2 Methodology, methods and analysis

### 2.1 Kaupapa Māori alignment

The Harti RCT is grounded in Kaupapa Māori Theory. This includes recognising the existence and validity of Māori knowledge, language and culture (12). Kaupapa Māori approaches to research have been described as “by Māori, for Māori, with Māori”, that Māori self-definitions and self-valuations are affirmed, as ensuring that tikanga Māori (practices and customs) and processes are followed throughout the research, Māori benefit from the study, and that colonial constructions and definitions of Māori are critiqued (13).

Kaupapa Māori Theory within the Harti programme recognises the need to critique conventional approaches, enact systems change, direct structural transformation; and address unequal power relationships to improve outcomes for Māori (12). Our study is underpinned by the He Pikinga Waiora (HPW) Implementation Framework—a theoretical and practical guide for developing and implementing interventions that has Māori self-determination at its core and includes co-design methods (14, 15). Co-design is an important best practice principle for Kaupapa Māori research (16). In part this is due to the iterative approach of co-design that allows for conceptual re-developments and refinement, based on the social-cultural needs of partnership groups (11). In line with the broader methodological Kaupapa Māori approach, the Harti process takes an ecological system-focused approach (17) to research questions, whereby our interest is understanding the complex environment, and supporting health outcome improvements at both the family (whānau) level as well as at the systems level, rather than a focus purely on individual patients’.

Holistic, whānau-centred care approaches have long been integral to Māori conceptualisations of health and wellbeing (18). The NZ Ministry of Health's Māori Health strategy He Korowai Oranga (2002) and Whakamaua: Māori Health Action Plan aim for a health system that will work in a way that acknowledges Māori health aspirations and the central role of whānau as a principal source of strength, support, security, and identity (19, 20), Family or whānau-centred practice is also being increasingly recognised as a key goal for modern healthcare. Whānau-centred practice is characterised by a “partnership between parents and service providers, a focus on the family's role in decision-making, and recognition that parents are the experts on their child” (p.357) (21). While the concepts of family-centred practice are often described within NZ's education, health, and social welfare sector policy documents (22), a disconnect has been identified between these descriptions of best practice and what in fact occurs (23).

### 2.2 Design

**Figure 1:**
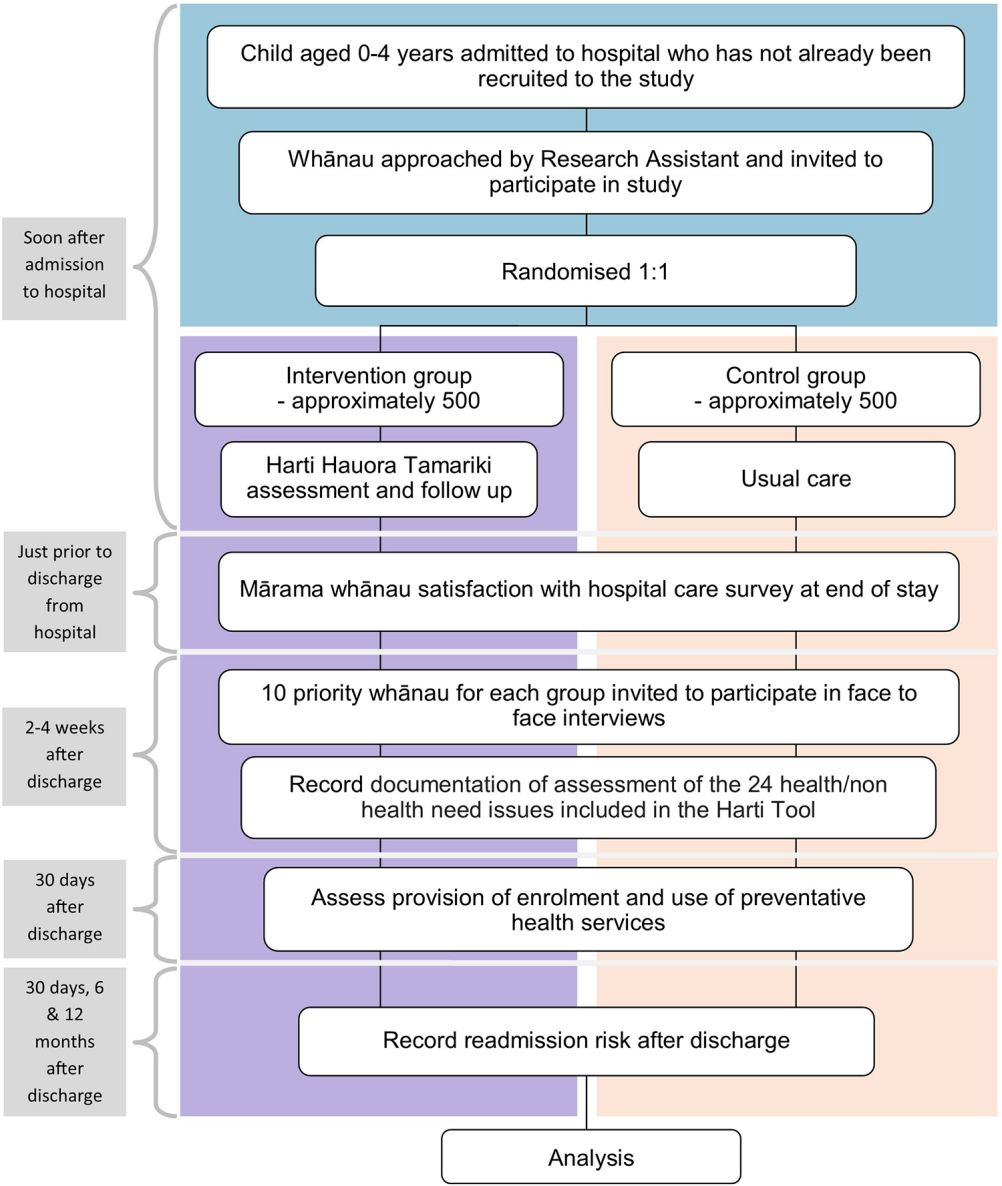
Schematic of trial design.

The study uses a Kaupapa Māori methodology with qualitative and quantitative methods. Qualitative methods include in-depth interviews with whānau and are published elsewhere (24). Quantitative methodology was a randomised controlled observer-blinded parallel groups single centre superiority trial. It is designed to measure the impact of the Harti tool, compared with usual hospital care, on child and whānau wellbeing outcomes, as specified in the primary and secondary endpoints. Tamariki and their whānau will be randomised to the intervention and control groups at a ratio of 1:1. The intervention group will receive usual care plus the Harti tool, and the control group will receive usual hospital care only, as per the trial schematic shown in Figure 1.

### 2.3 Intervention development

A co-design process was initiated as a response to issues of unmet health need and inequitable and unacceptable hospital readmission rates for tamariki Māori. The process was led by staff at Te Puna Oranga (the Māori Health Unit) at Te Whatu Ora Waikato, who worked with nurses, Kaitiaki (cultural support workers) and doctors who had worked or were working in child health at Waikato Hospital (11). The team identified that hospital care focused almost exclusively on single illnesses and that the model of care did not able the root causes of disease to be address, nor the broader opportunities to improve wellbeing for tamariki and their whānau to be realised. The team concluded that a holistic and whānau ora approach was needed for hospitalised tamariki and their whānau. Whānau ora describes the healthy families goal within New Zealand's Māori Health Strategy, He Korowai Oranga (25), and is also a government work programme. For this project we use the term whānau ora to describe an approach which places whānau at the centre of service delivery, requiring the integration of health, education, and social services around needs of whānau.

There is increasing recognition worldwide of the importance of wellbeing care needs (such as addressing poverty, education, housing, social and cultural cohesion) in policies and programmes aimed at improving health outcomes (26–30). There is also a developing understanding that the health care setting provides a unique opportunity to address both health and broader wellbeing care needs by improving the coordination and quality of health care; and more effectively supporting priority populations.

Over a 1 year period, the iterative co-design process resulted in the development of a tool to identity health and wellbeing needs, and to support opportunities to meet needs and enable the provision of holistic and preventative care particularly for hospitalised tamariki Māori and their whānau. The tool, developed for all tamariki hospitalised at Waikato Hospital, was named the Harti Hauora Tamariki Tool by the then General Manager of Te Puna Oranga, Māori Health. First iterations of the tool had sections for completion by both nursing and medical staff. However, the medical staff were not consistent in completion of their sections. The final iteration was completely a nursing document. The Harti tool was printed onto a card and included in the front of medical notes files. A staff training folder was developed, and training sessions were held with ward staff and a training folder was kept in each ward. A local Māori artist helped develop a whānau Harti information booklet with information about available services—which was given to all whānau along with a variety of resources for tamariki.

The Harti Tool was used in the paediatric medical wards at Waikato Hospital from 2014 to September 2017. The paediatric medical charge nurse reported that the majority (over 90%), of patients and their whānau were assessed using the tool during in that period. The Harti tool was used for the majority of tamariki and their whānau during this time.

#### 2.3.1 Redevelopment of the Harti tool and resources for the RCT

The Harti tool, staff training manual, and whānau booklet resources were redeveloped before the trial commenced (described below) and they will be revised and updated throughout the trial when required. The redevelopment is a necessary component that needs to occur before the trial to ensure that resources and pathways in the tool and staff training required is up to date and relevant for participants (i.e., localised to region and cohort).

For this redevelopment, the Harti study team were supported by an advisory and steering group that included whānau with experience of multiple hospital admissions with their tamariki. In accordance with the HPW framework, the team invited members with a range of expertise so that different understandings could be considered, and diverse perspectives could contribute to building a rich picture.

Over a 6-month period, redevelopment was guided by advisory and steering groups with influence sought and provided by whānau advisors who went through draft versions of the tool with the team and suggested changes at every step. Feedback was sought on the screening question, its relevant follow-up protocol, related training needs for staff, and whānau booklet content, barriers to ideal care and workable solutions, additional resources/brochures, or information to provide to whānau and measurable outcomes. In addition to providing relevant health information in a Māori centric format and entertain tamariki during their hospital stay, the whānau booklet had information included about the trial to inform families about Harti Hauora and related processes. Tool redesign included multiple meetings between the Project Manager and/or study investigators with subject matter experts (SME) for all topic areas in the tool. This included a facilitated wananga with local providers of support services for whānau that were included in the Harti tool.

**Figure 2:**
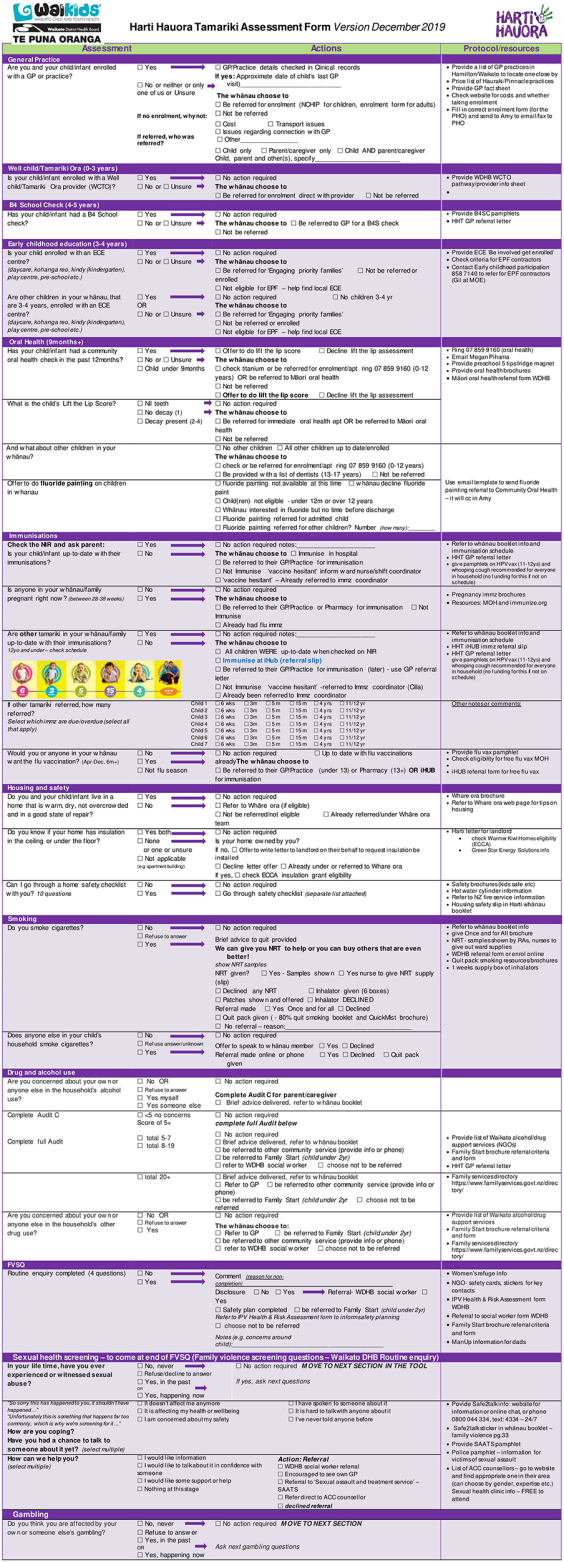
Final version of the Harti Hauora Tamariki Tool used in the trial.

After extensive feedback, the tool was revised and reviewed by steering committee members and the result was a 24-section screening assessment along with a staff training manual and whānau booklet. Table 1 shows the original topics included in the ward used tool alongside the additional 6 topics added during the redevelopment phase and to be used in the trial. See Figure 2 for tool contents and example of screening questions and follow up protocols.

**Table 1:** Original 18-items in Harti tool with additional 6 topics added for the trial version of the tool.

Original 18-item Harti tool	Topics added to the tool for the trial
Healthy homes assessment	Parenting support and resources
GP enrolment	Skin health
Childhood vaccinations	Gambling
Oral health enrolment	Social support and services
Household safety (accident prevention)	Drug and alcohol harm screening
Before school check (4-year-olds)	Vision and hearing
Wellchild Tamariki Ora	
Early childhood education	
Smoking cessation	
Family violence screening	
Access to car restraints	
Power to protect (shakin baby)	
Safe sleep	
Sore throat (rheumatic fever prevention)	
BMI	
Child development	
Breast feeding and support	
Frequent flyer screening	

**Figure:**
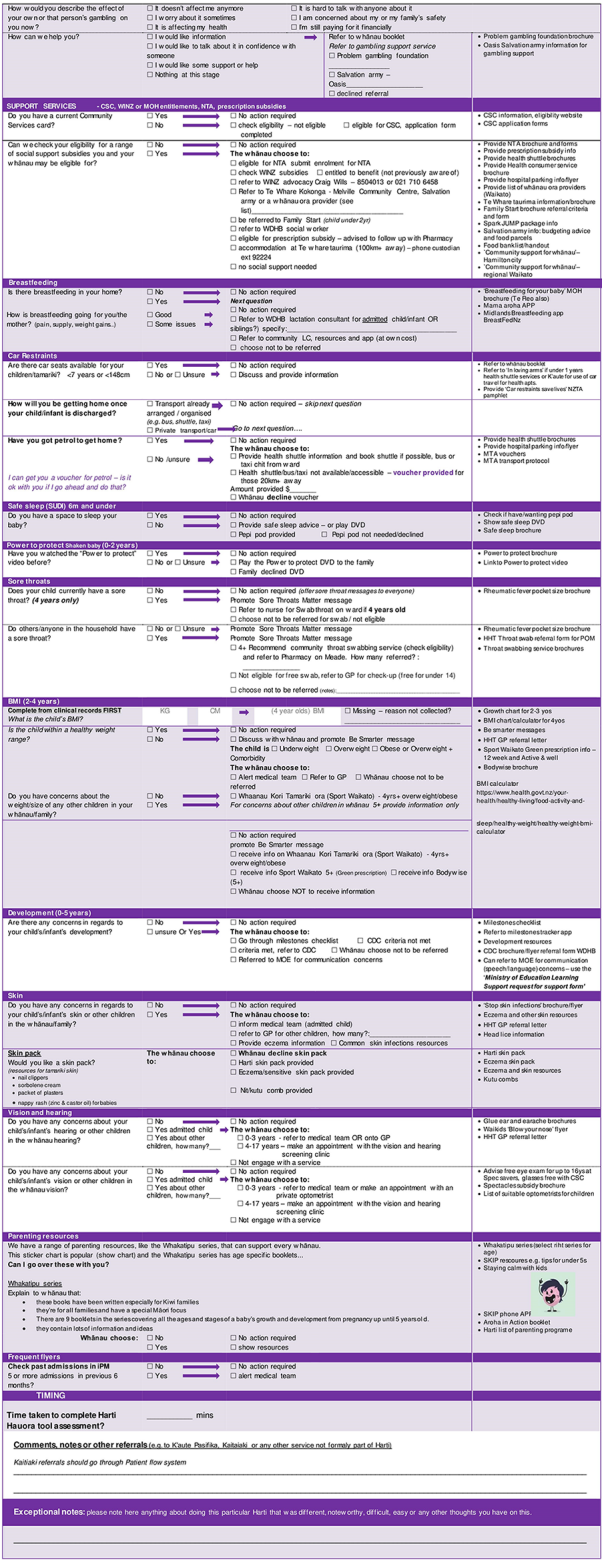


Research assistants (RA) were employed for their strengths in cultural safety and ability to engage with whānau Māori. Before the trial begins, training for RAs will take place over weeks with an emphasis on the importance of strong engagement with whānau. This includes learning about the Hui Process for engagement with Māori (31). As part of this, RAs are encouraged to take their time to engage with whānau and make every effort to build trusting relationships and whakamana or recognise the prestige of whānau. Staff engagement training also includes discussions on the importance of whānaungatanga, which is a practice of connecting and establishing respectful relationships. The importance of demonstrating manaakitanga, or showing respect, generosity and care will also be emphasised throughout training and will be revisited through the length of the project. These values will be embedded in the team at the start of the study and reiterated at every opportunity throughout the project including at team meetings, which will range from daily (during recruitment phases) to weekly in other phases.

### 2.4 Participant selection

#### 2.4.1 Inclusion criteria

Participants eligible for the Harti RCT are all patients currently residing in the Te Whatu Ora Waikato region in the central North Island of NZ, aged 0–4 years, with an acute medical admission to a paediatric ward at Waikato Hospital, Hamilton, Aotearoa NZ, in the period 3rd July 2018 to 19th December 2019.

An acute medical admission is defined as a patient with a length of stay greater than 3 h who is formally admitted to one of the Waikato Hospital's acute paediatric medical wards, or into the Kids Emergency Short Stay Unit, and whose admission is coded as acute (i.e., not arranged or elective) and whose admission was not for palliative care^1^. Only acute medical admissions will be eligible because the primary endpoint of the study is a reduction in the acute medical readmission risk. Waikato Hospital is the only recruitment site for feasibility and cost reasons. Several patients may be admitted to a paediatric ward and discharged before the research staff can approach them. This proportion is likely to be low and will be taken into consideration when interpreting findings.

The 0–4 age group is a focus of this research study for multiple reasons. Firstly, because of the special position of infants and children in Māori society as demonstrated by the whakatauki, or Māori proverb above. Important health need and ill health burden occurs during this period, with the 0–4 year old age group a significant policy and health service focus for the national health target on reduction of Ambulatory Sensitive (or preventative) Hospitalisations (ASH) in NZ (20). Important universal preventative care programmes exist for tamariki aged 0–4 years, such as early GP enrolment, immunisation, oral health care, and the Well Child/Tamariki Ora programme. Finally, this age group provides the greatest opportunity for critical and cost-effective early intervention across health, education, and social sectors. Focused investment on the early years is known to be critical for achieving health equity across the life course (32).

#### 2.4.2 Exclusion criteria

The study exclusion criteria included:

• Patients who have already entered the study
• Patients who are not eligible for publicly funded healthcare in New Zealand
• Arranged or wait list admissions to paediatric wards at Waikato Hospital
• Patients who are not a current resident in Te Whatu Ora Waikato region at the time of admission
• Patients with severe illness deemed by their medical team likely to die within 6 months of admission. This will be advised by the charge nurse who will provide a daily list of patients not to approach.

### 2.5 Baseline data collection

Recruitment and baseline data collection will be the responsibility of a RA who will be present, when possible, on the wards seven days per week. At the start of each day, a RA will generate a list of patients currently on the paediatric ward, screen them for eligibility, and check whether they have already been consented. RAs will check with the ward charge nurse to see if it is appropriate to approach the whānau of eligible patients. A record will be kept of all patients screened for eligibility to be approached and participate in the trial. Reasons for non-eligibility and/or refusal to participate will be recorded in a tracking sheet. Each RA will assure whānau that they have no obligation to provide a reason for non-participation in the trial. At the time of obtaining informed consent a baseline enrolment ascertainment form will be completed. The ascertainment form will record socio-demographic information (age, ethnicity and gender of tamariki patient and caregiver), method of transport to hospital, household size and structure, housing type and number of bedrooms, and level of material deprivation or NZiDep (33). The ascertainment form will be entered online using Qualtrics™ survey software.

### 2.6 Patient experience

A questionnaire on patient and whānau satisfaction with hospital experiences will be administered prior to discharge, or within 2 weeks of discharge. We will use the Mārama Real-Time Feedback tool, which is used extensively throughout health services in NZ. It was developed in 2016 by CBG Health Research as part of a one-year pilot funded by the Health and Disability Commissioner (HDC) (34).

### 2.7 Randomisation procedure

Randomisation will proceed according to a strict simple (non-stratified) protocol, following consenting, using the Qualtrics™ Survey software which uses the Mersenne Twister algorithm to determine allocation to intervention or control group. The RA will not reveal allocation to the participant, nor will anyone else. The only other person who will have access to group allocation data is the Study Manager.

The RA will continue with the Harti tool or inform usual care participants that they will return to go over the final short satisfaction with hospital experiences Mārama survey before they are discharged.

Because the intervention is whānau/family focussed, study contamination will occur if members of the same household are allocated to different intervention groups. Thus, for any child where another sibling or tamariki/child living in the same household has previously been consented and randomised in the trial, the subsequent child will be allocated to the same intervention group as their household member.

### 2.8 Sample size calculation

We aim to recruit a total of 1,000 participants, of whom we expect approximately 40% will be Māori (based on current figures and expected acceptability of the research protocol). This sample has been calculated to ensure that there is sufficient statistical power to detect a 7% absolute reduction in the 30-day readmission risk in the intervention group for all children and a 12% absolute reduction in the 30-day acute readmission risk for Māori. Sample size calculations are based on a two-tailed 0.05 level of significance with 80% power. We assume that the acceptability of participation will be similar for Māori and non-Māori.

We will measure differences between the intervention and control groups in the primary endpoint (readmission risk) controlling for age, sex, residing in Hamilton, socioeconomic deprivation (NZiDep), and RA responsible for conducting the intervention. Analyses of the primary endpoint will be stratified by ethnicity.

### 2.9 Informed consent

The adult/s accompanying the child and the child will be approached by the RA who will engage using the process of whānaungatanga and values of whakamana and manaakitanga. The RA will introduce herself/himself, and explain the study in broad terms, saying what participation may involve, including matching the child's National Health Index (NHI) number against health outcome databases, plus giving the opportunity to ask questions and give time to consider participation.

Whānau will be provided a brief verbal summary of the study, including the main aims and processes of enrolment. Those that do consent to taking part in the study will be asked to sign consent forms. Those that do not consent will receive usual hospital care. At the time of consent, parent/s/caregivers will be asked if any other children in the household have been consented into the study. If so, the child will be allocated to the same intervention group (as outlined above). Participants will be informed they can withdraw their child, and/or themselves from the trial at any time, but that analysis of collected data will be on-going.

### 2.10 Intervention procedure

The intervention will consist of a 24-section tool with screening questions and follow-up protocols for each question in accordance with the process shown in Figure 2. Intervention group patients and their whānau are invited to go over the Harti tool with the RA and provided with an information and activity booklet (whānau booklet). After engagement, RAs will go through the Harti tool on a tablet. RAs will read out questions in order and fill in answers for participants, in some cases participants may view questions and select responses themselves, if they prefer. The RA will follow established protocols to follow up on any identified areas of need. For example, if a child is not enrolled with a particular service, the RA will offer to assist with enrolment; if the patient or another child present or at home is identified to have a sore throat, then Rheumatic Fever prevention protocols will be followed. RA's will have equipment (cell phones and an iPad) to enable referrals and enrolments to take place at the bedside or in the interview room. The Harti RA will be trained in delivering high quality smoking cessation interventions for whānau and safe sleep devices including Pēpi Pods will be made available. If the whānau are not sure of the child's enrolment status (anecdotally a common occurrence), the Harti RA will match the patient’s National Health Index (NHI) number with selected databases and double check for access or check enrolment with the service over the phone or directly, e.g., oral health services.

### 2.11 Control group

This group will receive usual hospital care and be offered the Marama satisfaction with care survey at the end of their hospital stay.

### 2.12 Measures of effectiveness

A summary of each research hypothesis is listed in Table 2 and the details of how these will be examined is below.

**Table 2:** The primary (1, 1a) and secondary endpoint hypotheses summarised for the trial outcome data.

Hypothesis number	Hypothesis
1	Use of the intervention will result in a 7% lower readmission risk at 30-days for children aged 0–4 admitted to hospital for the intervention group compared to the usual care group.
1a	Use of the intervention will result in a 5% lower readmission risk at 30 days for children aged under 12 months admitted to hospital for the intervention group compared to the usual care group.
2	The intervention will result in a 5% higher satisfaction with hospital experience score for the intervention group compared to the usual care group.
3a	Compared to usual care, the intervention assessment will result in at least a 50% increase in the full documentation of non-enrolment with a GP, non-enrolment in oral health services, not up-to-date oral health checks, incomplete immunisation (for age) and incomplete WCTO participation.
3b	Compared to usual care, the intervention assessment will result in at least a 25% increase in full compliance with GP enrolment, oral health service enrolment, oral health checks, immunisation and WCTO participation.
4	Compared to the UC group, the intervention group will have at least a 25% higher complete smoking cessation referral rate for households with a documented resident who smokes.
5	The intervention group will have at least a 25% higher complete Whare Ora referral rate compared with the UC group
6	The intervention group will have a significantly lower median cumulative length of hospital stay in all acute admissions in the 12 months following discharge from the index admission event compared with the UC group
7	The readmission rate in the intervention group will be 25% less than the readmission rate in the UC group.

### 2.13 Primary endpoint

The primary endpoint is the relative risk of an acute hospital readmission in the 30 days following discharge for intervention group patients compared with control group patients (Hypothesis 1). The relative risk of readmission rather than readmission rate ratio was chosen as the primary endpoint because risk (probability of readmission within specified period) is easier to measure as it just requires counting the first time a patient is readmitted. By only counting the first readmission we avoid patients with large numbers of readmissions having a disproportion impact on the endpoint measure. This also simplifies statistical analysis. We have included the hazard ratio of readmission as a secondary endpoint (see below).

We will measure ethnic-specific differences in the primary endpoint measures and differences according to a 30-day^2^ readmission risk for Māori and non-Māori patients (combined).

To determine the superiority limit for the purposes of sample size calculations it was considered that the Harti programme should deliver at least a 7% absolute decrease in overall readmission risk as this would be both clinically meaningful for tamariki and whānau and financially meaningful for the hospital. Furthermore, we have designed the study so that it has power to detect a 12% absolute decrease in the 30-day risk of readmission for the Māori population. It is not powered to detect differences in risk of readmission between Māori and non-Māori tamariki.

### 2.14 Additional pre-specified analyses of an exploratory nature, based on the primary endpoint

These will include the following:

➢ The **hazard ratio** for (first) readmission in the intervention group compared with the control group. This compares time to the first readmission rather than just the fact of a readmission. As such, it can make use of varying lengths of follow-up time and may be more sensitive to the impact of the intervention. However, the use of the hazard ratio relies on the proportional hazards assumption, which is that the relative hazard of readmission remains constant over time.
➢ The **relative risk** of any readmission within 30 days of discharge, and from one to six months from the index admission event. This will examine whether the observed effect of the intervention on readmission rates occurs with a short or medium period. These analyses are exploratory as the trial has not been powered to detect difference within these time frames (although it may have adequate power to do so).
➢ A simple **Kaplan–Meir analysis** to compare the “survival” functions of intervention and control groups where survival in this case is taken to mean time following discharge without hospital readmission. For exploratory analysis of relative 6- and 12-month readmission risks this will also be used.
➢ Relative 30-day readmission risk in children aged less than 1 year at the time of their index admission (Hypothesis 1a). This outcome was added as a pre-specified analysis on the grounds that the interrupted time series analysis of the pilot suggested that the impact of the intervention is strongest (and possibly restricted to) this age group.

### 2.15 Secondary endpoints

There are seven quantitative secondary endpoints. Pre-testing will be undertaken to ensure that data can be accessed (e.g., GP enrolment and WCTO records) for all study endpoints.

### 2.16 Whānau satisfaction with care

**Figure 3:**
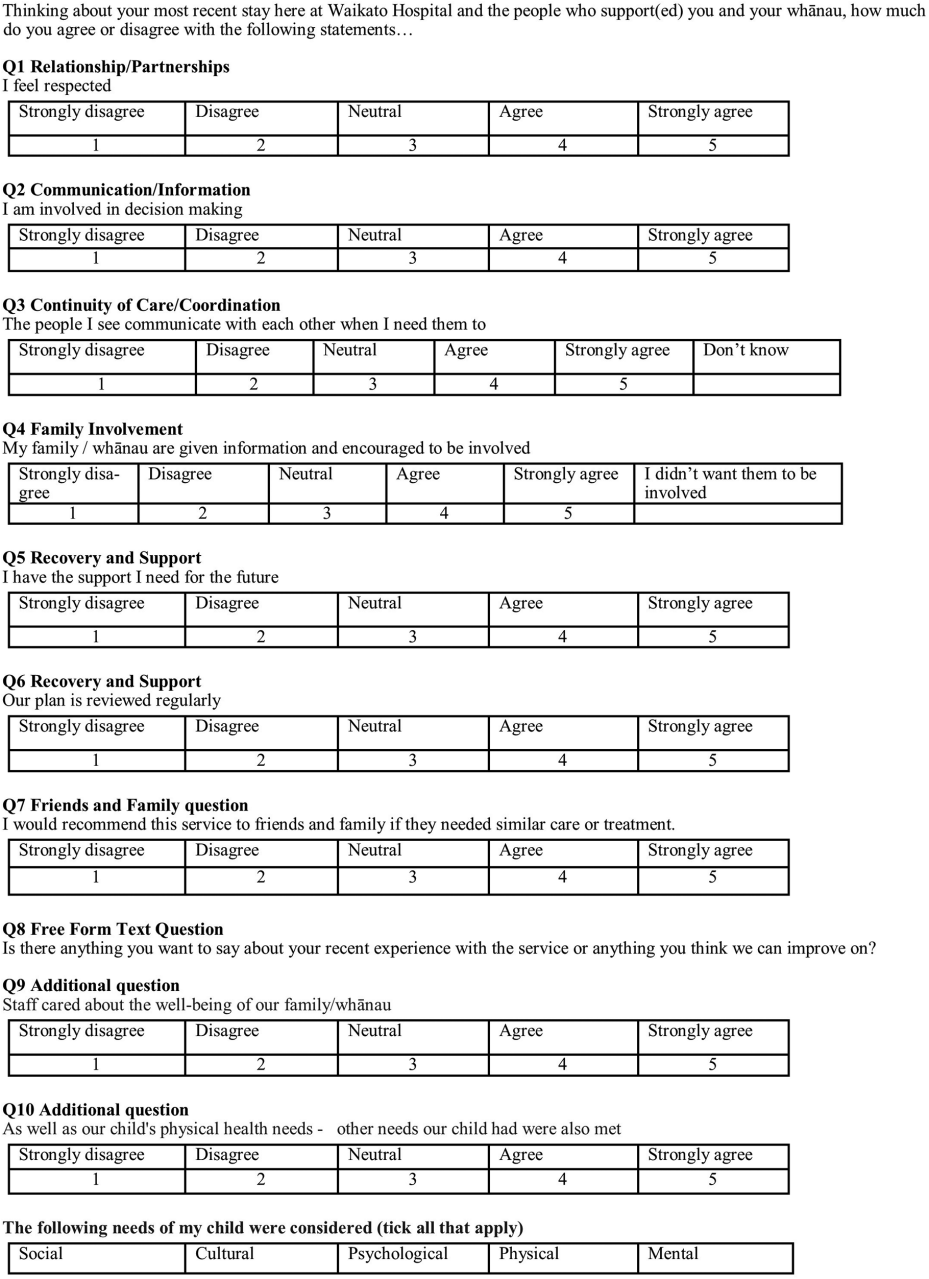
Patient satisfaction with hospital care (mārama survey).

Hypothesis 2 examines the difference in satisfaction with hospital experience between the intervention and control groups. This will be measured using the first seven questions from the Mārama real-time survey. Two questions have been added for the purposes of this research: one on the responsiveness to whānau needs, the other on whether a holistic approach was employed (Figure 3). An tenth open-ended Mārama question will also be asked and analysed. The responses will be scored on a 5-point Likert Scale ranging from Strongly Disagree to Strongly Agree (1 point for the former, 5 for the latter), so the total possible score range for someone answering all questions is from 9 to 45 which will be scaled to a range of 0–100 to accommodate cases where one or more questions is not responded to. The responses will be provided by the child’s primary care-giver.

### 2.17 Preventative health care

These hypotheses (3a & 3b) examine the difference in preventive services access and utilisation between the intervention and control groups. As previously mentioned, important universal preventative care is provided in the 0–4 year age group and for this reason these variables were chosen as secondary outcomes; general practice enrolment, oral health care, immunisations, and WCTO visits.

This endpoint has two parts: **documentation** and **achievement**. For documentation, external sources will be used to determine whether at admission the child was (i) enrolled with a General Practitioner (GP); (ii) enrolled with the oral health services; (iii) up to date with all oral health checks (seen in the last 12 months); (iv) up to date with all immunizations; (v) up to date with WCTO visits. The indicator that will be compared between the intervention and UC groups is the number of children with ‘non-compliance’^3^ in any of the five domains (GP enrolment, oral health enrolment, up-to-date oral health checks, up-to-date immunisation, and up to date WCTO visits). In whom compliance is achieved (fully documented in the intervention assessment or patient record during the child's admission), divided by the total number of children with non-compliance in any of these five domains.

For the **achievement** component of this endpoint (hypothesis 3b), which is contingent on detecting a significant difference in the **compliance** component, intervention and UC groups will be compared on the proportion of children who at admission were non-compliant on any of the five domains, and who became fully compliant within 30 days of discharge from the hospital. Fully compliant is defined as all of the following; enrolment with a GP, enrolment in oral health services, a completed or booked oral health check, complete on the pathway for immunisation (for age) and complete WCTO participation.

### 2.18 Tobacco smoking cessation

The immediate reduction of the number of people who smoke is of huge importance for Māori and one of the key health targets for the Ministry of Health NZ. Adult smoking rates for Māori are 21.3%, higher when compared with other ethnics groups such as Pacific (18.1%), and European/Other (7.9%) (35). It is clear that parental or household smoking increases children's risk of developing respiratory infections, which commonly result in hospital admissions (36).

For this endpoint (hypothesis 4), we will use the proportion of parent/caregivers where a referral to stop smoking services was made during the hospitalisation period for cessation services. This will be calculated by sending a list of parent/caregiver NHI numbers of all participant parent/caregivers to the sole face to face smoking cessation provider (Once and For All). The provider will then confirm if a referral had been received for that NHI number during the period of hospitalisation or within 30 days of discharge from hospital for the index admission.

### 2.19 Referral to the Whare Ora healthy homes service

It is well established that good housing is particularly important for the first 9-months of life (37). The Growing Up In New Zealand study showed that poor housing was strongly associated with adverse health outcomes, even when controlling for income (37). Healthy homes initiatives have been shown to reduce acute hospital admissions in a NZ setting (26). The Whare Ora programme is a Waikato Healthy Homes initiative that was developed in 2016 to help whānau to create healthier, warmer, safer and drier homes. The service is free to eligible whānau and provides healthy homes education, products such as curtains, heaters, dehumidifiers and draught stoppers, and referral of families for further services by other providers (38).

Hypothesis 5 examines the difference between intervention and UC groups in the proportion of children for whom a Whare Ora referral was made within 30 days post-discharge. This will be calculated simply as the number of Whare Ora referrals in each group divided by the total number of eligible households (as some participants live in the same household) in that group. Any household can be counted only once in the numerator.

### 2.20 Cumulative length of stay

Hypothesis 6 will compare differences between intervention and UC groups in median cumulative length of stay (LOS) in acute hospital admissions in the 12 months following discharge. Only coded events with lengths of stay of 3 h or more will be included in the calculation. Length of stay will be calculated in hours from the time of admission to the time of discharge using the hospital time stamp records. Length of stay will be calculated using, where appropriate, the rules established in the Ministry of Health's LOS indicator. These join events within the same districts where there is a transfer between the two and the second event commences less than 24 h after the end of the prior event. In these cases, if the initial admission is not acute then this and any length of stay in a joined subsequent hospitalisation, even if acute, will not be counted. Hospitalisations where none of the contributing stays is case-mix coded will also therefore be excluded, consistent with these rules. This endpoint will be calculated as the difference in median cumulative lengths of stay by individual (i.e., not summing for the group as a whole) and will include acute hospital stays at any hospital in the Waikato region. Using medians and individual level data will avoid outlier cases with very long lengths of stay having undue influence on the result of the test.

### 2.21 Frequency of readmission

The frequency of readmission is explored in Hypothesis 7. This endpoint will be calculated as the rate ratio of acute readmission in the 12 months following discharge from the index admission event. It differs from the primary endpoint in using all admissions in the numerator, and a person-time denominator, which will exclude any days in which the patient is in hospital.

### 2.22 Adverse events

The only adverse event data collected will be alive status within 12 months of index discharge.

### 2.23 Data handling and monitoring

Analysis will be performed on an intention to treat basis. That is, endpoints will be compared for the two groups as defined from the point of randomisation to intervention or control group. For the primary endpoint, participants who are readmitted multiple times from each group will be included in the analysis only for the first (valid) occasion on which they are readmitted in the 12 months following the index admission.

Bivariate analyses comparing groups will test for statistical significance using the Chi Square test for differences in proportions. For the length of stay secondary endpoint where a difference in medians is to be tested, the Mann–Whitney *U* test will be used.

Although the randomised design is expected to yield balanced intervention and control groups in a study of this size, logistic regression analysis will be used for the primary and secondary endpoints to control for important confounding variables and to facilitate the assessment of interaction variables that increase or decrease the efficacy of the Harti tool. Statistical significance will be tested at the alpha level of 0.05. We will control for the false discovery rate in the secondary endpoints using the Benjamini–Hochberg method (39).

The study is not powered to detect ethnic differences in the impact of the intervention (effect modification). However, we will nevertheless conduct exploratory tests for effect modification with ethnicity (Māori/non-Māori), gender (male/female excluding other), and deprivation (as a dichotomised variable) using the Breslow Day test for homogeneity of odds ratio, and standard maximum likelihood methods (likelihood ratio tests) based on binomial regression models. We will assume an additive interaction for these analyses and report the relative excess risk due to interaction (RERI) where this is significant effect.

### 2.24 Measures taken to avoid bias

There is a large risk of contamination or a spill over effect—meaning the control group could get some of the intervention. This could happen for example if the nurses caring for control patients included some of the Harti tool screening questions in their assessments—more so than they normally would. The Hawthorne effect could also contaminate the project, meaning that just having this project happening may lead to better assessment and documentation for control patients. To mitigate the effect of contamination we will remove all Harti tool resources from the ward and stop it's use 6 months before the study starts. We hope this will give enough time for staff to get used to not doing a Harti assessment and to forget the individual questions; there is a high turnover of staff on the wards also so many working during the trial will have never used the Harti tool. We are working to ensure that Harti tool questions are not included into the design of new patient assessment forms. We will explain that a study is taking place but not go into detail for staff and we will not mention the Harti tool. Instead, we will emphasise the patient satisfaction component of the study. During the study period, the RA will keep all documentation, even the blank forms—confidential so as not to contaminate the ward environment.

To maintain blinding and concealment, the only person on the ward who will know explicitly what status the child has, is the RA. The child and whānau may be able to guess from the intervention but will not be told explicitly which group they are in. Results of the intervention assessment will be kept off the ward. As all patients will be consented and given the whānau satisfaction questionnaire by the RA, other ward staff will see the RA sitting with every admitted child and whānau. They may be able to tell who gets an intervention because the RA will spend longer with those who get the intervention than s/he does with those who are randomised to the control group and just get consented and the Mārama satisfaction with care survey. However, contamination is not expected to influence outcomes unduly. The following roles/steps will be blinded to treatment allocation; the person reviewing the notes of control and intervention patients to assess documentation of evidence of assessment, treatment/service delivery and referral/enrolment; the person matching NHIs with service data-bases to assess enrolment and service use; the person matching NHIs with hospital readmission data.

### 2.25 Data management

Information provided by participants will be only accessible to members of the research team. Participant study files and all other information will remain strictly confidential, unless there is an immediate risk of serious harm to them or others. Participant information will be logged into a study tracking sheet. No potentially identifying information will be used in any reports on this study. Records will be stored for at least 5 years in a secure place at Te Whatu Ora Waikato after the completion of the study. All electronic records will be password protected and stored on a restricted access shared drive at Te Whatu Ora Waikato.

The Health and Disability Ethics Committee has determined that a Data Safety Monitoring (DSM) Committee is not required.

## 3 Discussion

The Harti development and research process aligns to Kaupapa Māori Theory through the prioritisation of Indigenous development and aspirations, the use of tikanga and mātauranga in the development of the intervention, and by our partnership approach with Māori and system stakeholders across the spectrum of programme design, implementation, and evaluation. At the same time our protocol aligns with the requirements of a robust RCT, and we have existing ethical approval and registration.

A universal approach to health care provision has failed to address ethnic inequities in Aotearoa New Zealand (40, 41). While the authors recognise that Māori-specific interventions are also necessary, and the universal versus targeted discussion an important one, in this RCT they wanted to test whether the Harti tool was an important addition in the paediatric ward for all children, as well as enhancing the outcomes specifically for Māori (on the background of greater need). As such, the potential for the findings of this proposed research to be translated into Māori health gains is high. Tamariki Māori have more to gain from the Harti programme than non-Māori children due to greater need, hence the Harti programme is expected to contribute toward decreasing the unjust and pervasive health inequities that exist between Māori and non-Māori New Zealanders, particularly for children. We aim to measure the opportunities for holistic care to reduce hospitalisation rates as our primary outcome, as well as a wide range of secondary outcomes and we have determined an appropriate sample size to ensure that we can understand the risk of hospitalisation as well as the rate of supports provided.

The need for Māori led holistic care that takes a whānau ora approach in addressing the determinants of health, health protective factors, and health risk factors is not limited to child health alone and the Harti approach could be used in multiple areas of Māori health need. In addition, while we aim to undertake a local intervention in the Waikato region of Aotearoa/New Zealand, there is important relevance for our processes and findings across regions and in a wide range of policy settings.

### 3.1 Ethics and dissemination

The RCT protocol has ethical approval from the Health and Disability Ethics Committee (HDEC), reference number: 18/CEN/88 and is registered with the Australian New Zealand Clinical Trials Registry (ANZCTR), registration number: ACTRN12618001079235. The Harti study has locality approval from the Waikato District Health Board Māori Ethics Review Committee (Ref: RD017074).

In accordance with the He Pikinga Waiora Framework, we will engage and work with our networks and seek guidance from leaders, and academic and coal face experts in the Māori health, health, policy/Ministry of Health, iwi health, and political spheres to develop integrated knowledge mechanisms including dissemination approaches. Throughout the research process we will update key stakeholders through established networks; these include regional child health action groups, and paediatric and Māori networks. Traditional academic dissemination techniques will also be used including publication in peer-reviewed journals, and presentation at national and international paediatric, and Māori health conferences. We will keep local Māori stakeholders abreast of our process and have built the development of a comprehensive stakeholder engagement and dissemination plan into our timeline and budget.

## Footnotes

^1^A revision was made early in the implementation to include the Kids Emergency short stay unit as many of those admissions were respiratory related (similar to ward admissions). This was a means of capturing ward admissions earlier for recruitment and to widen the pool of potential participants as many admitted to the ward were discharged before being approached for the study.

^2^The primary outcome readmission timeframe was changed from 12 months to 30 days before the trial was complete. This change was because we conducted an interrupted time series analysis of the piloting of Harti Hauora (before the trial began) in which it was noted that the strongest effect of the intervention was seen in 30 day readmission risk. The second reason for this change is that it will enable a shorter period of follow-up which will reduce the time required to report on the study findings, and also the costs of the study. This change was registered with the ANZCTR.

^3^Non-compliance (used in an entirely non-pejorative sense) is defined as: not being enrolled with a GP, not being up to date with immunizations, not being up-to-date with WCTO (including before school check) or not being up-to-date or enrolled with oral health checks.
